# Supplementary material for: Abundance of the vector Aedes aegypti in urban and rural areas in Managua, Nicaragua
Source: PLoS Negl Trop Dis. 2026 Apr 28;20(4):e0014256. doi: 10.1371/journal.pntd.0014256 (PMC13148774; doi:10.1371/journal.pntd.0014256)
Supplement: S1 Table — (DOCX) [file pntd.0014256.s001.docx]

**S1_Table. Demographic and household information and educational level of urban and rural households in District III of Managua, Nicaragua.**

| **Variables** | **Urban (%)** | **Rural (%)** |
| --- | --- | --- |
| **Demographic** |  |  |
| People in houses | 1,299 (53.4) | 1,132 (46.6) |
| People per house x̄ | 5.2 | 4.5 |
| **Age range** |  |  |
| ≤ 5 years | 163 (12.5) | 119 (10.5) |
| 6-17 years | 361 (27.8) | 303 (26.8) |
| 18-59 years | 670 (51.6) | 630 (55.7) |
| >60 years | 105 (8.1) | 80 (7.1) |
| **Respondent sex** |  |  |
| Female | 223 (89.2) | 206 (82.4) |
| Male | 27 (9.8) | 44 (17.6) |
| **Educational level** |  |  |
| No school studies | 17 (6.8) | 14 (5.6) |
| Some studies | 159 (63.6) | 141 (56.4) |
| High school completed | 51 (20.4) | 64 (25.6) |
| University | 23 (9.2) | 31 (12.4) |
| **Public utilities** |  |  |
| **Waste management** |  |  |
| Burn waste | 12 (4.8) | 120 (48) |
| Bury waste | 1 (0.4) | 8 (3.2) |
| Municipal waste collection service | 217 (86.8) | 82 (32.8) |
| Throw waste in vacant lots | 2 (0.8) | 24 (9.6) |
| Throw waste into water channels | 18 (7.2) | 16 (6.4) |
| **Water Supply in houses** |  |  |
| Maintained uninterrupted water supply | 163 (65.2) | 122 (48.8) |
| Faced interruptions in water supply | 87 (34.8) | 128 (51.2) |
| Daily interruptions in water supply | 78 (89.7) | 108 (84.3) |
| Mean daily water interruption hours | 11 | 13 |
| **Electricity Supply in houses** |  |  |
| Houses with electrical access | 243 (99.1) | 235 (96.7) |
| Houses without electrical access | 2 (0.8) | 8 (3.3) |
| No data | 5 | 7 |
| With an electricity meter at the house | 163 (67.0) | 112 (47.7) |
| **Houses sanitation** |  |  |
| Flush toilet in house | 175 (71.4) | 167 (69.0) |
| Houses with pit latrines | 66 (26.9) | 70 (28.9) |
| Houses with a flush toilet and latrine | 4 (1.6) | 5 (2.0) |
| No data | 5 | 8 |
| **Internet Access** |  |  |
| Houses with internet service | 43 (17.6) | 37 (15.2) |
| Houses without internet service | 202 (82.4) | 206 (84.8) |
| No data | 5 | 7 |
| **Cooking practices** |  |  |
| Use firewood for cooking | 158 (64.4) | 186 (76.5) |
| No data | 5 | 7 |
| **Frequency of firewood use** |  |  |
| Use firewood to cook daily | 32 (20.3) | 48 (25.8) |
| Use firewood to cook weekly | 94 (59.5) | 97 (52.2) |
| Use firewood to cook Bi-weekly | 17 (10.8) | 24 (12.9) |
| Use firewood to cook monthly | 15 (9.4) | 17 (9.1) |
| **Houses structure** |  |  |
| **Type of flooring** |  |  |
| Concrete floor | 87 (34.8) | 67 (26.8) |
| Cement tiles | 18 (7.2) | 30 (12.0) |
| Dirt floor | 58 (23.2) | 76 (30.4) |
| Ceramic floor | 54 (21.6) | 47 (18.8) |
| Dirt and concrete | 13 (5.2) | 18 (7.2) |
| Concrete and ceramic | 20 (8.0) | 12 (4.8) |
| **Type of wall** |  |  |
| Concrete | 152 (60.8) | 120 (48.0) |
| Concrete and wood | 26 (10.4) | 69 (27.6) |
| Concrete and zinc | 14 (5.6) | 13 (5.2) |
| Concrete and Cement Sheet | 12 (4.8) | 3 (1.2) |
| Wood | 6 (2.4) | 17 (6.8) |
| Wood and zinc | 5 (2.0) | 4 (1.6) |
| Zinc | 33 (13.2) | 21 (8.4) |
| Cement Sheet | 2 (0.8) | 1 (0.4) |
| Mud brick | 0 (0) | 2 (0.8) |
| **Type of roof** |  |  |
| zinc roof | 250 (100) | 249 (99.6) |
| Fiber-cement roof | 0 (0) | 1 (0.4) |
