# Supplementary material for: Abundance of the vector Aedes aegypti in urban and rural areas in Managua, Nicaragua
Source: PLoS Negl Trop Dis. 2026 Apr 28;20(4):e0014256. doi: 10.1371/journal.pntd.0014256 (PMC13148774; doi:10.1371/journal.pntd.0014256)
Supplement: S2 Table — (DOCX) [file pntd.0014256.s002.docx]

**Table S2. Entomological collections of *Ae. aegypti* larvae in urban and rural settings of Managua, Nicaragua, during dry and rainy seasons of 2022 and 2023.**

| **Study site** | **Season** | **Year** | **L1** | **L2** | **L3** | **L4** |
| --- | --- | --- | --- | --- | --- | --- |
| **Rural** | Dry | 2022 | 108 | 206 | 955 | 2034 |
|  | Rainy | 2022 | 543 | 1325 | 1451 | 4365 |
|  | Dry | 2023 | 685 | 1057 | 1219 | 1477 |
|  | Rainy | 2023 | 853 | 1837 | 2833 | 5098 |
|  |  | *Total* | *2,189* | *4,425* | *6,458* | *12,974* |
| **Urban** | Dry | 2022 | 123 | 263 | 336 | 510 |
|  | Rainy | 2022 | 484 | 1114 | 638 | 1341 |
|  | Dry | 2023 | 174 | 290 | 425 | 819 |
|  | Rainy | 2023 | 358 | 633 | 1092 | 2609 |
|  |  | *Total* | *1,139* | *2,300* | *2,491* | *5,279* |
