# Supplementary material for: Abundance of the vector Aedes aegypti in urban and rural areas in Managua, Nicaragua
Source: PLoS Negl Trop Dis. 2026 Apr 28;20(4):e0014256. doi: 10.1371/journal.pntd.0014256 (PMC13148774; doi:10.1371/journal.pntd.0014256)
Supplement: S3 Table — (DOCX) [file pntd.0014256.s003.docx]

**S3_Table. Entomological collections of adult *Ae. aegypti* in urban and rural settings of Managua, Nicaragua, during dry and rainy seasons of 2022 and 2023.**

| **Study site** | **Season** | **Year** | **Females** | **Males** |
| --- | --- | --- | --- | --- |
| **Rural** | Dry | 2022 | 33 (7.5%) | 48 (10.8%) |
|  | Rainy | 2022 | 142 (32.2%) | 158 (35.6%) |
|  | Dry | 2023 | 87 (19.7%) | 78 (17.6%) |
|  | Rainy | 2023 | 179 (40.6%) | 160 (36.0%) |
|  |  | *Total* | *441 (100%)* | *444 (100%)* |
| **Urban** | Dry | 2022 | 18 (6.6%) | 22 (7.1%) |
|  | Rainy | 2022 | 58 (21.2%) | 87 (28.2%) |
|  | Dry | 2023 | 45 (16.4%) | 59 (19.1%) |
|  | Rainy | 2023 | 153 (55.8%) | 141 (45.6%) |
|  |  | *Total* | *274 (100%)* | *309 (100%)* |
