# Supplementary material for: Abundance of the vector Aedes aegypti in urban and rural areas in Managua, Nicaragua
Source: PLoS Negl Trop Dis. 2026 Apr 28;20(4):e0014256. doi: 10.1371/journal.pntd.0014256 (PMC13148774; doi:10.1371/journal.pntd.0014256)
Supplement: S4 Table — (DOCX) [file pntd.0014256.s004.docx]

**S4_Table. House index (HI)**

| **Study site** | **Season-Year** | **Total houses** | **Positive houses** | **HI** |
| --- | --- | --- | --- | --- |
| Rural | DS^a^ 2022 | 250 | 53 | 21.2% |
| Urban | DS 2022 | 250 | 44 | 17.6% |
| Rural | DS 2023 | 250 | 116 | 46.4% |
| Urban | DS 2023 | 250 | 60 | 24.0% |
| Rural | RS^b^ 2022 | 250 | 148 | 59.2% |
| Urban | RS 2022 | 250 | 107 | 42.8% |
| Rural | RS 2023 | 250 | 147 | 58.8% |
| Urban | RS 2023 | 250 | 103 | 41.2% |

^a^DS, dry season; ^b^RS, rainy season.
