# Supplementary material for: Abundance of the vector Aedes aegypti in urban and rural areas in Managua, Nicaragua
Source: PLoS Negl Trop Dis. 2026 Apr 28;20(4):e0014256. doi: 10.1371/journal.pntd.0014256 (PMC13148774; doi:10.1371/journal.pntd.0014256)
Supplement: S5 Table — (DOCX) [file pntd.0014256.s005.docx]

**S5_Table. Container index (CI)**

| **Study site** | **Season-Year** | **Total containers** | **Positive containers** | **CI** |
| --- | --- | --- | --- | --- |
| Rural | DS^a^ 2022 | 943 | 62 | 6.6% |
| Urban | DS 2022 | 604 | 51 | 8.4% |
| Rural | DS 2023 | 1,047 | 186 | 17.8% |
| Urban | DS 2023 | 659 | 87 | 13.2% |
| Rural | RS^b^ 2022 | 785 | 284 | 36.2% |
| Urban | RS 2022 | 608 | 171 | 28.1% |
| Rural | RS 2023 | 1,294 | 418 | 32.3% |
| Urban | RS 2023 | 787 | 188 | 23.9% |

^a^DS, dry season; ^b^RS, rainy season.
