# Supplementary material for: Abundance of the vector Aedes aegypti in urban and rural areas in Managua, Nicaragua
Source: PLoS Negl Trop Dis. 2026 Apr 28;20(4):e0014256. doi: 10.1371/journal.pntd.0014256 (PMC13148774; doi:10.1371/journal.pntd.0014256)
Supplement: S6 Table — (DOCX) [file pntd.0014256.s006.docx]

**S6_Table. Breteau index (BI)**

| **Study site** | **Season-Year** | **Total houses** | **Positive containers** | **BI** |
| --- | --- | --- | --- | --- |
| Rural | DS^a^ 2022 | 250 | 62 | 24.8 |
| Urban | DS 2022 | 250 | 51 | 20.4 |
| Rural | DS 2023 | 250 | 186 | 74.4 |
| Urban | DS 2023 | 250 | 87 | 34.8 |
| Rural | RS^b^ 2022 | 250 | 284 | 113.6 |
| Urban | RS 2022 | 250 | 171 | 68.4 |
| Rural | RS 2023 | 250 | 418 | 167.2 |
| Urban | RS 2023 | 250 | 188 | 75.2 |

^a^DS, dry season; ^b^RS, rainy season.
