# Supplementary material for: Abundance of the vector Aedes aegypti in urban and rural areas in Managua, Nicaragua
Source: PLoS Negl Trop Dis. 2026 Apr 28;20(4):e0014256. doi: 10.1371/journal.pntd.0014256 (PMC13148774; doi:10.1371/journal.pntd.0014256)
Supplement: S7 Table — (DOCX) [file pntd.0014256.s007.docx]

**S7_Table. Pupae per houses index (PHI)**

| **Study site** | **Season-Year** | **Total**  **houses** | **Total**  **Pupae** | **PHI** |
| --- | --- | --- | --- | --- |
| Rural | DS^a^ 2022 | 250 | 270 | 1.08 |
| Urban | DS 2022 | 250 | 57 | 0.23 |
| Rural | DS 2023 | 250 | 466 | 1.86 |
| Urban | DS 2023 | 250 | 249 | 1.00 |
| Rural | RS^b^ 2022 | 250 | 966 | 3.86 |
| Urban | RS 2022 | 250 | 228 | 0.91 |
| Rural | RS 2023 | 250 | 1,260 | 5.04 |
| Urban | RS 2023 | 250 | 503 | 2.01 |

^a^DS, dry season; ^b^RS, rainy season.
