# Supplementary material for: Abundance of the vector Aedes aegypti in urban and rural areas in Managua, Nicaragua
Source: PLoS Negl Trop Dis. 2026 Apr 28;20(4):e0014256. doi: 10.1371/journal.pntd.0014256 (PMC13148774; doi:10.1371/journal.pntd.0014256)
Supplement: S8 Table — (DOCX) [file pntd.0014256.s008.docx]

**S8_Table. Pupae per containers index (PCI)**

| **Study site** | **Season-Year** | **Total containers** | **Total Pupae** | **PCI** |
| --- | --- | --- | --- | --- |
| Rural | DSa 2022 | 943 | 270 | 0.29 |
| Urban | DS 2022 | 604 | 57 | 0.09 |
| Rural | DS 2023 | 1,047 | 466 | 0.45 |
| Urban | DS 2023 | 659 | 249 | 0.38 |
| Rural | RSb 2022 | 785 | 966 | 1.23 |
| Urban | RS 2022 | 608 | 228 | 0.38 |
| Rural | RS 2023 | 1,294 | 1,260 | 0.97 |
| Urban | RS 2023 | 787 | 503 | 0.64 |

^a^DS, dry season; ^b^RS, rainy season.
