# Supplementary material for: Abundance of the vector Aedes aegypti in urban and rural areas in Managua, Nicaragua
Source: PLoS Negl Trop Dis. 2026 Apr 28;20(4):e0014256. doi: 10.1371/journal.pntd.0014256 (PMC13148774; doi:10.1371/journal.pntd.0014256)
Supplement: S9 Table — (DOCX) [file pntd.0014256.s009.docx]

**S9_Table. Pupae per persons index (PPI)**

| **Study site** | **Season-Year** | **Total persons** | **Total Pupae** | **PPI** |
| --- | --- | --- | --- | --- |
| Rural | DS^a^ 2022 | 1,105 | 270 | 0.24 |
| Urban | DS 2022 | 1,286 | 57 | 0.04 |
| Rural | DS 2023 | 1,119 | 466 | 0.42 |
| Urban | DS 2023 | 1,263 | 249 | 0.20 |
| Rural | RS^b^ 2022 | 1,174 | 966 | 0.82 |
| Urban | RS 2022 | 1,320 | 228 | 0.17 |
| Rural | RS 2023 | 1,129 | 1,260 | 1.12 |
| Urban | RS 2023 | 1,328 | 503 | 0.38 |

^a^DS, dry season; ^b^RS, rainy season.
