# Supplementary material for: Abundance of the vector Aedes aegypti in urban and rural areas in Managua, Nicaragua
Source: PLoS Negl Trop Dis. 2026 Apr 28;20(4):e0014256. doi: 10.1371/journal.pntd.0014256 (PMC13148774; doi:10.1371/journal.pntd.0014256)
Supplement: S10 Table — (DOCX) [file pntd.0014256.s010.docx]

**S10_Table. Adult index (AI)**

| **Study site** | **Season-Year** | **Total houses** | **Positive houses** | **AI** |
| --- | --- | --- | --- | --- |
| Rural | DSa 2022 | 250 | 47 | 18.8% |
| Urban | DS 2022 | 250 | 35 | 14.0% |
| Rural | DS 2023 | 250 | 80 | 32.0% |
| Urban | DS 2023 | 250 | 63 | 25.2% |
| Rural | RSb 2022 | 250 | 128 | 51.2% |
| Urban | RS 2022 | 250 | 87 | 34.8% |
| Rural | RS 2023 | 250 | 133 | 53.2% |
| Urban | RS 2023 | 250 | 127 | 50.8% |

^a^DS, dry season; ^b^RS, rainy season.
