# Supplementary material for: Abundance of the vector Aedes aegypti in urban and rural areas in Managua, Nicaragua
Source: PLoS Negl Trop Dis. 2026 Apr 28;20(4):e0014256. doi: 10.1371/journal.pntd.0014256 (PMC13148774; doi:10.1371/journal.pntd.0014256)
Supplement: S11 Table — (DOCX) [file pntd.0014256.s011.docx]

**S11_Table. Adults per house (AH)**

| **Study site** | **Season-Year** | **Total houses** | **Total**  **Adults** | **AH** |
| --- | --- | --- | --- | --- |
| Rural | DS^a^ 2022 | 250 | 81 | 0.32 |
| Urban | DS 2022 | 250 | 40 | 0.16 |
| Rural | DS 2023 | 250 | 165 | 0.66 |
| Urban | DS 2023 | 250 | 104 | 0.42 |
| Rural | RS^b^ 2022 | 250 | 300 | 1.20 |
| Urban | RS 2022 | 250 | 145 | 0.58 |
| Rural | RS 2023 | 250 | 339 | 1.36 |
| Urban | RS 2023 | 250 | 294 | 1.18 |

^a^DS, dry season; ^b^RS, rainy season.
