# Supplementary material for: Abundance of the vector Aedes aegypti in urban and rural areas in Managua, Nicaragua
Source: PLoS Negl Trop Dis. 2026 Apr 28;20(4):e0014256. doi: 10.1371/journal.pntd.0014256 (PMC13148774; doi:10.1371/journal.pntd.0014256)
Supplement: S12 Table — (DOCX) [file pntd.0014256.s012.docx]

**S12_Table. Adults per person (AP)**

| **Study site** | **Season-Year** | **Total persons** | **Total Adults** | **AP** |
| --- | --- | --- | --- | --- |
| Rural | DS^a^ 2022 | 1,105 | 81 | 0.07 |
| Urban | DS 2022 | 1,286 | 40 | 0.03 |
| Rural | DS 2023 | 1,119 | 165 | 0.15 |
| Urban | DS 2023 | 1,263 | 104 | 0.08 |
| Rural | RS^b^ 2022 | 1,174 | 300 | 0.26 |
| Urban | RS 2022 | 1,320 | 145 | 0.11 |
| Rural | RS 2023 | 1,129 | 339 | 0.30 |
| Urban | RS 2023 | 1,328 | 294 | 0.22 |

^a^DS, dry season; ^b^RS, rainy season.
