# Supplementary material for: Abundance of the vector Aedes aegypti in urban and rural areas in Managua, Nicaragua
Source: PLoS Negl Trop Dis. 2026 Apr 28;20(4):e0014256. doi: 10.1371/journal.pntd.0014256 (PMC13148774; doi:10.1371/journal.pntd.0014256)
Supplement: S13 Table — (DOCX) [file pntd.0014256.s013.docx]

**S13_Table. Females per person (FP)**

| **Study site** | **Season-Year** | **Total persons** | **Total Females** | **FP** |
| --- | --- | --- | --- | --- |
| Rural | DS^a^ 2022 | 1,105 | 33 | 0.03 |
| Urban | DS 2022 | 1,286 | 18 | 0.01 |
| Rural | DS 2023 | 1,119 | 87 | 0.08 |
| Urban | DS 2023 | 1,263 | 45 | 0.04 |
| Rural | RS^b^ 2022 | 1,174 | 142 | 0.12 |
| Urban | RS 2022 | 1,320 | 58 | 0.04 |
| Rural | RS 2023 | 1,129 | 179 | 0.16 |
| Urban | RS 2023 | 1,328 | 153 | 0.12 |

^a^DS, dry season; ^b^RS, rainy season.
