# Supplementary material for: Abundance of the vector Aedes aegypti in urban and rural areas in Managua, Nicaragua
Source: PLoS Negl Trop Dis. 2026 Apr 28;20(4):e0014256. doi: 10.1371/journal.pntd.0014256 (PMC13148774; doi:10.1371/journal.pntd.0014256)
Supplement: S14 Table — (DOCX) [file pntd.0014256.s014.docx]

**S14_Table. Fixed-effect estimates from a generalized linear mixed model evaluating factors associated with female per person rates of *Aedes aegypti* abundance.**

| **Variable** | **Exp (β)** | **β (SE)** | **95% CI** | **Z value** | **p-value** |
| --- | --- | --- | --- | --- | --- |
| Season (Rainy) | 3.23 | 1.171 (0.185) | 2.25 – 4.63 | 6.33 | <0.001 |
| Year (2023) | 2.1 | 0.743 (0.129) | 1.63 – 2.71 | 5.75 | <0.001 |
| Community (Rural) | 1.67 | 0.511 (0.114) | 1.33 – 2.09 | 4.48 | <0.001 |
| Pupae abundance | 1.04 | 0.0358 (0.0048) | 1.03 – 1.05 | 7.43 | <0.001 |
| Water-holding containers | 1.05 | 0.0441 (0.0127) | 1.02 – 1.07 | 3.46 | <0.001 |
| Average humidity | 0.98 | −0.0167 (0.0073) | 0.97 – 1.00 | −2.30 | 0.021 |
