# Supplementary material for: Abundance of the vector Aedes aegypti in urban and rural areas in Managua, Nicaragua
Source: PLoS Negl Trop Dis. 2026 Apr 28;20(4):e0014256. doi: 10.1371/journal.pntd.0014256 (PMC13148774; doi:10.1371/journal.pntd.0014256)
Supplement: Attachment — Submitted filename: Response to reviewers Final.docx [file pntd.0014256.s016.docx]

**Additional Editor Comments:**

Dear Dr. Suazo-Laguna and colleagues,
Thank you for your submission to PLOS Neglected Tropical Diseases. Your manuscript, investigating Aedes aegypti densities in rural versus urban settings, addresses an understudied topic with direct relevance to public health in the region and beyond. Both reviewers recognize the importance of your findings.
After careful consideration of the reviews, I am pleased to inform you that we have made a decision of “minor revision” on your manuscript. The key results are sound and relevant. However, the reviewers had some suggestions that, if addressed, will significantly strengthen the clarity, contextualization, and impact of your paper.
Below is a summary of issues raised by the reviewers that should be addressed:
Both reviewers emphasized the need to clearly define and contextualize the urban and rural classifications. Please provide a more detailed description of the physical and infrastructure differences between the settings. A table contrasting features (e.g., housing density, drainage, garbage collection, household spacing, presence of screens or gutters, vegetation, etc.) and/or representative photographs would help clarify.
Reviewer 1 would like more detail on the spatial distribution of sampled houses. Please consider including a map that indicates all houses surveyed (not just those with adult mosquito detections) and comment on the proximity of houses relative to Aedes aegypti dispersal ranges.
Reviewer 2 suggested enhancing the clarity and impact of your findings related to container productivity and Stegomyia indices. Please consider including per-house estimates in the container productivity table and revisiting the description and implications of the indices reported in Figure 3 (including adding index names to the figure itself)
Reviewer 2 found the manuscript overly verbose at times. Please revise for conciseness and stronger narrative focus, particularly in the results and discussion sections. Highlight the key findings and emphasize the implications for vectorial capacity.
Please ensure clarity in your methods regarding:
• Which larval stages were counted (e.g., only L4 and pupae?)
• The absence (or presence) of Ae. albopictus
• Aspirator model (change to “Prokopack” as per R2)
• Index definitions—adding a table for clarity is encouraged
Reviewer 1 encouraged the sharing of per-household data (with identifying details removed) and the inclusion of spatial data if possible. We strongly encourage making this information available in supplementary materials or in an open repository to enhance the value of your work for reuse and meta-analysis.
Please consider addressing the following:
• Seasonal patterns and inter-annual variation in mosquito density
• Potential roles of vegetation or ornamental plants as breeding habitats
• Non-residential breeding sites and their role in sustaining Ae. aegypti populations
Please revise your manuscript to address the above points and provide a detailed point-by-point response to each reviewer's comment. Be sure to indicate where changes have been made in the manuscript text.
We look forward to receiving your revised manuscript. Please do not hesitate to contact me if you have any questions or need clarification on the requested revisions.

 	Response to editor:

We sincerely thank you and the reviewers for the constructive feedback and thoughtful comments on our manuscript. We have carefully reviewed each point raised and provided detailed responses and revisions throughout the manuscript. In particular, we have enhanced the clarity of our definitions of urban and rural settings, improved figure presentation, included additional spatial and contextual data, and streamlined the Results and Discussion sections for conciseness and stronger narrative focus. We have also expanded methodological details and ensured that figure legends and tables are clearer and more informative. We have made efforts to provide as much clarity and contextual comparisons between urban and rural sites. We hope these revisions address all of the reviewers’ concerns and strengthen the overall clarity, contextualization, and impact of the manuscript. We truly appreciate the reviewers’ insights and the opportunity to improve our work.

Reviewer #1: The survey methodology was rigorous and well planned but limited in scale (only 2 years and a total of 4 surveys). Statistical analysis appears appropriate, and I have no ethical concerns. No meteorological analysis was attempted, although I accept that two surveys were conducted during the dry season and two surveys during the wet season. Some description of average rainfall totals for the two seasons would be useful context.

**Response:** In response to the Reviewer’s suggestion, we have provided a description of the average rainfall and temperature for the two seasons in the Materials and Methods on Line 104-105: “In 2022, the mean surface air temperature was 25.8°C with 2,438 mm of precipitation, while in 2023, it was 26.2°C with 2,234 mm of precipitation (23)”.

Reviewer #2: In this paper, Suazo-Laguna et al explore the density of adults and larvae in rural vs urban residences (houses/yards). It is highly impressive with ~1000 houses and highlights that Aedes aegypti (an extremely important disease vector) numbers were higher in rural areas then the authors expected (although I note believe, it would help to better convey to readers in this Nicaragua context, what is urban and what is rural)
I think this is a good manuscript with important findings.

I provide a number of specific and more general comments below to help improve the manuscript.

Specific Comments:

Line 20: consider adding yellow fever

**Response:** We have added yellow fever to the text.

Line 109: is it “classification system” ? Plural “classifications? “

**Response:** We have adjusted the text to “classification system”, as suggested.

Line 109: I expand this in my point 1 below, but what is the definition of urban and rural? I think it would help to spell it out or picture it, etc. From a global level I would imagine wh-at is urban and what is rural is highly subjective and varies by country.

**Response:** We have moved the definition of *urban* and *rural* from the introduction to the Materials and Methods to the methods section (lines 110–114), which now reads:

“In Nicaragua, the definition of *urban* is any locality that is a departmental, regional, or municipal capital, or any population center with ≥1,000 inhabitants that also has basic urban infrastructure (i.e., a laid-out street grid, electricity supply, and commercial or industrial establishments). *Rural* areas are defined as those that do not meet these criteria and include dispersed populations living outside defined settlements (25).”

In addition, we revised Figure 1 to illustrate the contrast between the two categories with representative street-view photographs. We also generated a Supplemental Table S1 (Additional file 1: Table S1) to provide a clearer comparison of the characteristics of the study sites classified as urban versus rural. (lines 220-229)

“We did observe statistical differences between sites in relation to public utilities such as waste management (burn waste: χ2=120.1, p<0.001; bury waste: χ2=5.5, p=0.02; municipal waste collection: χ2=151.6, p=<0.001; throwing waste in vacant lots: χ2=19.64, p<0.001), water supply (uninterrupted water supply: χ2=13.72, p<0.001), electricity supply (formal house meter: χ2=20.7, p<0.001) and type of wall construction (concrete: χ2=8.3, p=0.004). Generally, rural sites relied more on burning waste (48% vs 5%), had lower access to municipal waste collection services (32% vs 87%), faced higher interruptions in water supply (51% vs 35%) and lacked formal electricity meter connected to the power grid (48% vs 67%) compared to urban sites. In Nejapa, the mean number of building structures within a 150-m2 grid was 46.5 (SD=24.9) compared to 123.7 (SD=41.6) in Camilo Ortega.”

Line 135: “the MOH also carried out” ?

**Response:** To provide more clarity on this matter we have adjusted the text (lines 139-141), which now reads:

“As part of standard procedures, the MOH also conducts routine Ae. aegypti control and surveillance activities across all districts of Managua, which could have affected some of the study households.”

Linew 135-141 : Was this at same houses? Or in the district, in general to some of the houses?

**Response:** The activity is conducted in the same district, in the general area where the houses our found. We provide further clarity in the text to avoid confusion on Lines 139-145:

“As part of standard procedures, the MOH conducts routine *Ae. aegypti* control and surveillance activities across all districts of Managua, which could have affected some of the study households. Every two months, MOH vector control field technicians visit houses to eliminate immature vectors by placing larvicide in clean water containers in the house. Complementary activities carried out included pesticide fumigation and community clean-up campaigns. To minimize interference, we scheduled our activities to avoid overlapping with MOH vector control efforts.”

Line 185: Breteau index, Citation?

Response: We have added the following citation 31:

PAHO. Métodos de vigilancia entomológica y control de los principales vectores en las Américas [Internet]. Pan American Health Organization; 2021 [cited 2025 Oct 8]. Available from: https://iris.paho.org/handle/10665.2/55241

Line 217 “.R -space’ code” ?

**Response:** We have improved the clarity of where to find the code repository. Line 206 now reads:

“For a detailed step-by-step procedure, see the “Availability of data and material” section for the link to our code repository.”

Availability of data and materials

Data set is available in ZENODO: <https://doi.org/10.5281/zenodo.14861397> and R code can be found at: <https://github.com/jgjuarez/UrbanRuralManagua>

Line 432- 433: How close were your houses? Aedes is not thought to travel very far (perhaps as low as ~100 meters in their lifetime) Does that change interpretation?

**Response:** House selection was conducted with a minimum spacing of 50 m between households, although actual distances varied by community. In rural areas, households were often located up to 30 m farther apart than those in urban settings. While *Aedes* mosquitoes are generally thought to have limited flight ranges of 50–100 m, studies in rural settings have reported dispersal distances of up to 200 m. Based on this, we believe that even with a conservative dispersal estimate of 100 m, there would be sufficient dispersal of the mosquito population to neighboring households. Within approximately three generations (spanning from the dry to the rainy season), this dispersal would likely allow coverage of about half of the community, driven by these key sites. Nonetheless, we have revised our interpretation to place greater emphasis on the importance of area-wide management strategies.

Lines 99-131/ Figure 1 : See above, here and/or Line 432-433 please consider discussing how close your collections houses are to each other/ their density. Is it possible to have a map of the collection sites showing appx locations of the collection sites ? It is unclear to me from Fig 1 all the collection sites since it only shows where adults were found - not all the sampling places.

**Response:** We have generated a new Figure 1 that shows the distribution of the households without revealing their true location. The legend now reads:

“Figure 1. Entomological collections conducted in dry and rainy seasons of 2022-2023 in communities of District III of Managua, Nicaragua. A) Rural (Nejapa) and Urban (Camilo Ortega) communities of District III. Black dots indicate the approximate distribution of study households. Geographic coordinates were randomly offset to obscure exact household locations. B) Street view of a typical household structure and adjacent road in Nejapa. C) Street view of typical household structures and adjacent road in Camilo Ortega.”

Line 433-434 : see above comment, Aedes control can be hyper-local (at the house level) to make a difference. My concern is that what you actually have to do is find the key spots, but only area-wide management is going to locate these places.

**Response:** We appreciate the Reviewer’s thoughtful comment. We agree with the Reviewer that while control can be highly effective at the household level, area-wide management is essential for reliably identifying and addressing these key sites. We have revised the text to better reflect this point, particularly considering the patterns emerging from our rural study areas. A more detailed treatment of these findings will be presented in a forthcoming manuscript. Lines 409-416:

“We also acknowledge that this study did not evaluate non-residential breeding sites (i.e., schools, cemeteries, churches, tire shops, etc.), which can also play a role in sustaining mosquito populations in the area. We are currently evaluating the impact of non-residential sites as drivers of population dynamics in District III. Of note, in the 2023 rainy season, we recorded for the first time adult *Ae. albopictus* within 46 houses (urban: 13; rural: 33) of District III, showcasing the need for further surveillance efforts to evaluate the impact that *Ae. albopictus* might have in the ecology of *Ae. aegypti* and the transmission of arboviruses.”

Line 469-472: Can you share some examples of types of non-residential locations one/ you might/are consider investigating?

**Response:** We have provided clarity into the structures we are considering evaluating on line 410:

“(i.e. schools, cemeteries, churches, tire shops, etc.),”

Line 472: Perhaps note how many occurrences (e.g. how many houses were they found? ) to give an idea of establishment of this species?

**Response:** We have provided the information regarding the total number of houses on line 414:

“we recorded for the first time adult *Ae. albopictus* in 46 houses (urban: 13; rural: 33) of District III”

Figure 3: The figure would be enhanced for ease of reader by listing the measure (e.g. House Index (HI) in the figure itself, instead of / in addition to the figure legend.

**Response**: We have adjusted the figure and added the measure in the figure itself, in addition to the figure legend.

Other questions and points:
1. Could some pictures of typical residences or breeding sites be provided in rural / urban context? Would that help? I’m thinking about Lines 223-236 where you note the demographic structure of the communities, but understanding the physical landscape differences of the structures, properties, etc would be nice. How “developed” (or not) are these. Do houses tend to have drains/ Gutters? Is trash in yard common, or not? {information on trash collection services might further contextualize this). Do houses tend to be screened or not? How close (generally) is one house to another in rural vs urban?

**Response:** We adjusted Figure 1 to showcase the typical residence in rural/urban context. Now Figure 1 legend reads as:

“B) Street view of a typical household structure and adjacent road in Nejapa. C) Street view of typical household structures and adjacent road in Camilo Ortega.” Additionally, we have generated a new Table S1 and expanded the Results section regarding physical landscape features and structures to better showcase key differences between the sites.

2. It would seem mosquito numbers went up from 2022 to 2023? Is it worth noting any thoughts on why?

**Response:** We did observe an increase in mosquito numbers from 2022 to 2023 across both urban and rural areas during the dry season of 2023. In contrast, during the rainy season, this increase was only evident in urban areas. We suspect that this could be related to social practices of water storage since 2023 had overall less precipitation than 2022, which resulted in higher water intermittency in 2023. We recognize this as a limitation, likely influenced by the availability of only two time points for evaluation. Another scenario is that vector control by the MoH could have occurred closer to our surveillance points during the rainy season of 2022, impacting mosquito abundance. Unfortunately, we do not have information regarding the exact date of vector control procedures in the sites.

3. Did you consider vegetation as larval source? Some plants can be larval sites in them self (e.g. bromeliads) Also it has been reported that irrigation etc for the purposes of maintaining decorative gardens led to increased larval density in ‘wealthier’ parts of town. Do your urban residents keep plants? Are they un-unidentified sources of larva?

**Response:** We did not explicitly consider vegetation (e.g., bromeliads) as a larval source. However, we did account for flowerpots and other plant-related containers. While we did identify them as sources of larvae, the numbers varied substantially, and therefore we grouped them into the broader “useful container” category. To clarify this, we have updated the legend of Figure 2 to explicitly indicate the inclusion of flowerpots. Interestingly, we did observe that almost 10% of *Ae. albopictus* (even though we didn’t find many during this period) were collected from flowerpots.

Results

Reviewer #1: Yes overall, but the text of the article should be streamlined and reduced considerably. There is a lot of text and descriptive work without telling a story. Take home about Aedes densities being higher is rural than urban locations is clear, but not a lot more to say here.

**Response:** We have worked on the Results and Discussion sections to streamline the text to better reflect our currents results.

Reviewer #2: yes

Conclusions

Reviewer #1: Overall, yes, but the presentation should be far more concise. Found much of the text, did not have a clear point being made, and more effort to contrast the two sites are needed beyond stating that one is rural and the other urban based on National definitions. Both sites seem urban to me.

**Response:** We have edited the manuscript to provide more clarity in each section and to clarify our results in these communities. Additionally, we have made changes to more clearly differentiate between urban and rural communities.

Reviewer #2: yes

Data presentation:

Reviewer #1: (No Response)

Reviewer #2: Data presentation:

I would argue that the authors should be able to share per-residence data of each measure collected. I am assuming this would not have GPS / address data. This would improve reuse.

if some sort of spatial information (such as obfuscated GPS points could be provided, that would be even better.)

**Response:** We have generated new obfuscated GPS points for the spatial sampling, with random allocation at 100m from the original site. In addition, we have edited Figure1 as requested by Reviewer 1 to better showcase the sampling distribution of the households. We have additionally, generated obfuscated GPS points and uploaded them to the Zenodo repository. [10.5281/zenodo.14861396](https://d.docs.live.net/48322b5fca08d482/Berkeley%20and%20SSI/Manuscritos/A2CARES/Urban%20rural%20Harold/PlosNTD/2nd/10.5281/zenodo.14861396)

Reviewer #1: General Comments
1. The findings that Aedes aegypti densities are higher in rural compared to urban communities is important and few comparisons have been published. I would, however, suggest that the manuscript be streamlined to focus on this point.

**Response:** We have adjusted the text to streamline the focus on this point through the manuscript.

2. The methodology was rigorous and analysis appropriate, but the study was rather small, covering two years and two sites only. I recognize this cannot be changed but this represents only four surveys, albeit done well. Again, this information is valuable, but this manuscript needs to emphasize differences between the rural versus urban sites, which appears to be principally during the rainy season where there are more (useless containers).

**Response:** We have generated a new Supplementary Table S1 that showcases the differences between urban and rural. We mainly observe a difference between urban and rural areas in relation to how services are provided or accessed, such as waste management, water intermittency, and formal electricity service. We further expand on this matter in the main text in the Results on lines 220-229:””

“We did observe statistical differences between sites in relation to public utilities such as waste management (burn waste: χ2=120.1, p<0.001; bury waste: χ2=5.5, p=0.02; municipal waste collection: χ2=151.6, p=<0.001; throwing waste in vacant lots: χ2=19.64, p<0.001), water supply (uninterrupted water supply: χ2=13.72, p<0.001), electricity supply (formal house meter: χ2=20.7, p<0.001) and type of wall construction (concrete: χ2=8.3, p=0.004). Generally, rural sites relied more on burning waste (48% vs 5%), had lower access to municipal waste collection services (32% vs 87%), faced higher interruptions in water supply (51% vs 35%) and lacked formal electricity meter connected to the power grid (48% vs 67%) compared to urban sites. In Nejapa, the mean number of building structures within a 150-m^2^ grid was 46.5 (SD=24.9) compared to 123.7 (SD=41.6) in Camilo Ortega.”

3. The issue of urban versus rural is not very convincing here, as the population in the “rural” area is a little less than half of the urban area. We need a better comparison which probably exists. Items, like housing density and infrastructure, would provide much needed context. If the comparison of the data used from satellites would be useful. The figures are very nice but don’t give a strong contrast to the naked eye. One of the major problems here is that there is a minimal and no statistical difference in the community demographics of the two communities.

**Response:** As mentioned in the previous response, we have added a Supplementary Table S1 to showcase differences between rural and urban communities with a Chi^2^ statistical analysis to compare rural and urban communities. We have expanded the Results to comment on the difference observed between urban and rural sites and incorporated some more information regarding house density by community in the methodology, lines 228-229: “In Nejapa, the mean number of building structures within a 150 m^2^ grid was 46.5 (SD=24.9) compared to 123.7 (SD=41.6) in Camilo Ortega.”

4. Methods: It is not clear from the methods what immature stages were being counted. Was it only 4th instar larvae and pupae? Also you mention Ae. albopictus in the methods but no mention after that. If no Ae. albopictus were found state it there.

**Response:** We counted all larval instars as well as pupae present in containers. To clarify, we have revised the Methods (line162) to read: *“*with all immature stages counted and included in the analysis*.”*

Regarding *Ae. albopictus*, we encountered 344 larvae and 73 pupae in houses, almost exclusively during the rainy season of 2023. Given the relatively low numbers, we did not emphasize these findings in the results; however, the presence of adults is noted in the Discussion (line 413).

5. In various places, proportions/percentages are given in ranges but it is not clear if this is between years or another spatial unit. There is mention of 21 neighborhoods, but I don’t think it applies here.

**Response:** We apologize for the confusion; the comparison is between years and seasons. We have revised the text to address this issue throughout the manuscript.

6. Note for the Container productivity, also refer to the table and simply describe broader trends. Something like: “Overall we collected 35% more containers and more than double pupae positive containers in rural sites compared to urban sites. Add a column for the per house estimates of the number of containers.

**Response:** We have adjusted the text as suggested by the Reviewer to provide broader trends, for example, on Lines 234-235: “We observed that 60% of all containers were in rural sites, which also yielded more than twice as many pupae per positive container compared to urban sites (Table 2).” We also have adjusted the table to add the column per house estimate of the number of containers.

7. The Stegomyia indices reported in Figure 3 are quite high overall, even for the urban communities. Compare this to Line 254-257, when 18-46% of the houses had at least one container with larvae. Looking at the distribution might better capture this. Overall, I would spend more text describing Figure 3 than you do on container productivity.

**Response:** We have re-edited the Results to better reflect the comments of the Reviewer and have made abundant changes throughout the text to better capture the nature of our data and the widespread abundance of *Ae. aegypti* in both urban and rural settings, underscoring the results for rural communities.

8. Presentation of model results. Could large trends be presented – that is overall, rainy season had higher indices that during the dry season; rural higher than urban; then you can point out differences between years, but we are most interested in main effects.

**Response:** We now present the larger trends for the model and have edited the text as follows on lines (295-304):

“However, we observed distinct patterns of mosquito infestation and abundance between urban and rural communities. The GLMM indicated that a Negative Binomial type 2 distribution provided the best fit (AIC=4408.1). Overall, mosquito abundance showed clear large-scale trends: higher during the rainy season compared to the dry season, higher in rural communities compared to urban ones, and higher in 2023 compared to 2022. Specifically, the model estimated that the rainy season was associated with more than a two-fold increase in female *Ae. aegypti* abundance compared to the dry season (OR = 2.82, 95% CI: 2.4–3.3, p < 0.001). Rural communities consistently had higher mosquito abundance, with urban sites containing 36.5% fewer females (OR = 0.635, 95%CI 0.53-0.77, p< 0.001). Across years, abundance in 2023 was significantly higher than in 2022 (OR = 1.72, 95% CI: 1.46–2.02, p < 0.001).”

9. Although I agree that the literature does emphasize urban transmission of dengue by its vector Ae. aegypti, the presence of this vector in rural locations is not new, especially in Asia. The western hemisphere is a bit different because the species was eradicated in the 1960-70s and probably became reestablished in urban areas first and now expanding to other areas.

**Response:** We have edited the discussion to further showcase the rural presence, *Ae. aegypti* in Asia and Africa, and how this contrast to urban data from Latin America.

Specific Comments
Line 162. Change to using Prokopack aspirators (these are not backpack aspirators).

**Response:** We have made the adjustment as suggested by the Reviewer.

Line 182: Suggest you make a table of the indice definitions.

**Response:** We have made a Table 1 for the indice definitions.

Line 223-236. This would be better presented in a table (it is in supplementary material, but I don’t seem to have access), but again let the reader see the table and highlight the differences which there does not appear to be.

**Response:** We have generated a Supplementary Table S1 that shows key differences for urban and rural sites. We expanded the comments regarding key differences observed in the analysis.

In addition, we revised Figure 1 to illustrate the contrast between the two categories with representative street-view photographs. We also generated a Supplemental Table S1 (Additional file 1: Table S1) to provide a clearer comparison of the characteristics of the study sites classified as urban versus rural (lines 220-229)

Line 254-261. Not clear what the take hope is, this observation is best show with a House Indext or Breteau index. We know that Ae. aegypti distributions are very clustered. How do your statements have implications for vector surveillance.

**Response:** We have adjusted the text to better reflect our observation that even though mosquito distributions are clustered, there are some key homes that could ultimately have a larger impact if control activities were carried out specifically in those sites. We also clarify the importance of area-wide approaches for vector control. Lines 387-391

“We also observed that a small subset of houses disproportionately produced pupae, consistent with hotspot dynamics reported elsewhere (48-62). However, adult captures were more evenly distributed, likely reflecting dispersal from hotspots (63). This suggests that while hotspot elimination may reduce larval productivity, area-wide approaches remain essential for controlling mobile adult populations.”

Line 268-270. This information should go into a table that contrasts urban versus rural communities, although it seems like the excess production in rural communities is mostly rain-filled containers, possibly more useless containers than in urban settings?

**Response:** Yes, we have included a table that showcases these results and have expanded our comments on these results. See lines 247-256:

“In urban communities, the containers most productive for pupae, regardless of year and season, were barrels (55-79%), followed by buckets (0.4-32%) and, to a lesser extent, cement laundry sinks (0%-9%) (Figure 2). In rural communities, barrels were again the most productive containers (39-65%), though a greater diversity of container types (useful and non-useful containers) contributed substantially during the rainy seasons, accounting for up to 40% of pupal productivity. It is key to highlight the importance of water intermittency in the region. Our results show that 34.8% of urban houses and 51.2% of rural houses faced interruptions in water supply; of those with daily interruptions, urban houses experienced an average of 11 hours (SD=5.77) and rural houses 13 hours (SD=7.77) per day without running water, respectively. This has been previously shown to be a risk factor for immature Aedes presence in houses (15).”

Line 276: Again clarify are we talking about L4s only.

**Response:** We counted all larval instars as well as pupae present in containers. To clarify, we have revised the Methods (line162) to read: *“..*all immature stages were counted and included in the analysis*.”*

Line 346. Don’t understand the title here nor the discussion.

**Response:** We have tried to provide an adjusted title that better underscores our results and edit the discussion of them. The changes are found on lines (329-343), which now reads:

“*Non-linear relationships between pupal abundance and adult female Ae. aegypti…*”

We evaluated the non-linear relationship (smooth terms) between immature stages (L4 and pupae) and the abundance of female *Ae. aegypti*. Among the candidate models, pupal abundance provided the best fit for predicting female abundance (AIC = 4599.8), with a statistically significant smooth term (edf = 3.48, χ² = 46.07, p < 0.001; R² adj = 0.126). By contrast, the effect of total water-holding containers was not statistically significant (p = 0.09), although a positive linear trend suggested that more containers were generally associated with more adult females. When modeled by community, distinct patterns emerged. In rural communities, the relationship between pupal counts and female abundance showed a threshold-like pattern: an initial increase in adult females with 1–18 pupae, a plateau between ~19–41 pupae, and a sharp rise beyond 42 pupae. Predicted female counts increased from 0.24 (95% CI = 0.20–0.29) at 1 pupa to 0.49 (95% CI = 0.36–0.66) at 13 pupae, remained stable up to ~41 pupae, and then rose to 2.03 females (95% CI = 0.35–11.83) at 84 pupae (Figure 6). This suggests the presence of pupal thresholds in rural settings that may drive sharp increases in adult abundance once exceeded that are not observed in urban communities.”

Reviewer #2: (No Response)
