# Supplementary material for: Abundance of the vector Aedes aegypti in urban and rural areas in Managua, Nicaragua
Source: PLoS Negl Trop Dis. 2026 Apr 28;20(4):e0014256. doi: 10.1371/journal.pntd.0014256 (PMC13148774; doi:10.1371/journal.pntd.0014256)
Supplement: Attachment — Submitted filename: Reviewer Response Final.docx [file pntd.0014256.s017.docx]

**Response to Reviewers**

We thank the reviewers for their continued thoughtful and constructive feedback. We appreciate the time invested in evaluating our manuscript and believe the revisions have substantially strengthened the clarity, rigor, and interpretability of the study. Below, we respond point-by-point to Reviewer #4’s comments and clarify how the manuscript has been revised accordingly. Minor editorial suggestions from Reviewer #1 have also been addressed.

**Methods**


Reviewer #1: Overall, previous feedback has been considered and the missing study descriptions provided, although I do not see the supplemental information directly. Based on reviewer responses I'm satisfied.

Only minor point here is the larval counting. You have clarified in the reviewer responses that you counted all stages (not usually done for productivity) but I would explicitly state that since it is unusual. I would also like some feedback about what the distribution of 1st, 2nd, 3rd, and 4th instars were. Not the focus of the manuscript but when you say you counted all instars, one wants to know what the distribution is.

**Response:** We agree with the Reviewer and have now explicitly stated in the additional material Supplementary Table S2 the total counts of all immature stages for L1-L4.

Reviewer #3: (No Response)

Reviewer #4: The field methods are robust. I have concerns with the statistical analyses.

Much of the manuscript relies on what I would consider global comparisons - chi-square tests of raw values/counts across a few high-level categories. The authors admit there is "significant" (significance is undefined) heterogeneity in larval/pupal collections among houses in both areas and in both seasons; they yet then implement statistical tests that assume independence and homogeneity of variance. For instance, the authors admit that some containers were sampled multiple times (repeated measures violate independence). I'm also not following how global indices (such as those listed in table 1 which list the total sample size as a denominator) can be compared statistically - are there perhaps subunits of collections within each district? If so, then perhaps statistical tests make sense - if not, then its simply a descriptive comparisons.

**Response:** We thank the reviewer for their comments. We have made the following substantive revisions:

1. *Stegomyia* and pupal indices are now presented descriptively only, without inferential comparisons across communities.
2. All inferential analyses now rely on mixed-effects modeling frameworks that explicitly account for:
   - Repeated measures at the household level
   - Observer variability
   - Spatial clustering
3. Random intercepts for household (HouseID) and surveyor (Surveyor) were incorporated in all GLMM/GAMM models.

These changes are described clearly in the revised Data Analysis section.

So much per household information is washed out (for instance, lines 203 - 205 indicate zero values were removed), I really wonder that if such heterogeneity was actually analyzed, if the differences would stand between "rural" and "urban".

**Response:** We would like to clarify that zero values were not removed from any analysis. The full distribution of counts (including zeros) has been always retained. Container productivity modeling focused on pupae (as specified), not because zeros were excluded, but because pupae better predict adult emergence. The manuscript text has been revised for clarity. Line 201-203 “Container productivity analysis was only evaluated for *Ae. aegypti* pupae, as it has been shown that this entomological indicator is the most accurate to translate to adult abundance”. We have also added a Panel A to Figure 2 to show the percentage of the total containers found.

The linear vs. non-linear comparisons I think needs to be redone. The listed package gamm4 can compare linear and non-linear terms which I would recommend the authors consider. Then, AIC scores are more directly comparable and the notion of "thresholds" would be more statistically valid (pending non-linear models actually out perform linear models). I'd also consider BIC and RMSE type values be considered in model development/comparison. Also, some sort of assessment for zero-inflation is also likely needed given descriptions of collections provided by the authors.

**Response:** We fully agree with the Reviewer and have substantially strengthened the modeling framework. Specifically, we evaluated Poisson and zero-inflated model structures using the **glmmTMB** package, as gamm4 does not support zero-inflated formulations. Zero-inflated Poisson and negative binomial models were fitted and compared against standard Poisson mixed-effects models using AIC, BIC, and residual diagnostics (on this approach and the previous one). Zero-inflation did not improve model performance and was therefore not retained in the final analysis.

We acknowledge that direct comparisons between glmmTMB and spline-based GAM frameworks (e.g., mgcv::gam) are not strictly equivalent, particularly when random-effects parameterization differs. However, published methodological evaluations have demonstrated comparable performance of these frameworks for count and zero-inflated count data under similar conditions (see link below). While this comparison extends beyond the central objectives of our study, it provides additional reassurance that zero-inflation does not materially influence our inference.

https://www.r-bloggers.com/2017/05/fitting-count-and-zero-inflated-count-glmms-with-mgcv-2/

We would also like to comment that the following has also been expanded on the code repository:

1. GLMM (linear pupal term) and GAMM (smoothed pupal term) were directly compared in gamm4 using:
   - AIC
   - BIC
2. The GAMM provided improved model fit (AIC 2846 vs 2855), supporting non-linearity.
3. Zero-inflated Poisson and negative binomial models were evaluated using glmmTMB. Zero-inflation did not improve model performance and was therefore not retained.
4. Dispersion statistics and residual diagnostics were assessed.
5. The random structure (household + surveyor) is now explicitly stated.

Since the authors admit there is high heterogeneity of collections, raw GLMM/GAMM tables should be provided - I could not access the listed code/data availability (perhaps user error in broken links). Its also unclear if GLMMs represent any sort of model selection outcome (forward/backward/stepwise) or if they assess interactions or if they represent a full model of additive terms or individual unimodal glmm comparisons of each value. Finally, authors should state what the random terms were.

**Response:** In the previous analysis, no model selection was performed since the main effects were all significant. In this version, we have expanded our analysis to other environmental, social and climatic factors. In this manuscript, we intentionally avoid the inclusion of interaction terms to reduce complexity in our findings and overall interpretation. In this new analysis, model selection was based on Backward elimination using AIC/BIC.

Only statistically supported terms were retained.

The final GAMM included:

- Smoothed pupal abundance
- Season
- Year
- Community
- Total water containers
- Humidity

**Results**


-Does the analysis presented match the analysis plan?

-Are the results clearly and completely presented?

-Are the figures (Tables, Images) of sufficient quality for clarity?

Reviewer #1: Vast improvement, you might consider in Table 3 (adults also including adults/household - although you do describe this in the text).

Your efforts to streamline the manuscript have greatly paid off - much better manuscript.

Reviewer #3: (No Response)

Reviewer #4: The original reviewers identified a need to further identify how one region was urban and the other was rural. I would consider edits in response of this criticism insufficient. The authors mention they use categories defined by the MOH with rural sites being deficient in something - provided results clearly show the "rural" site as deficient but its never explicitely stated exactly how the rural region is distinguished from the urban. Seeing all of the data and amendments to the text, this feels more like a socio-economic gradient rather to a land cover gradient. Rural will be conflated with agrarian/farming and low density - all of the data presented suggest this is more of a comparison between urban/suburban type populations with some areas having infrastructure and other areas not having infrastructure. That would make more sense that aegypti is more prevalent in spaces that lack infrastructure. Perhaps that is less flashy of a result, but it would still be important to reinforce to a global audience - perhaps there is room for discussing the nuances of urban/rural classifications when it comes to a global vector?

**Response:** We appreciate the Reviewer’s thoughtful perspective regarding the interpretation of rural–urban classifications. Our intention was not to redefine rurality using alternative metrics but rather to evaluate mosquito ecology within the framework currently used by the Nicaraguan Ministry of Health (MOH), which defines the operational classification of communities for surveillance and control activities. A previous reviewer commented on this topic, and we expanded the definition to clarify the classification. Because vector control programs and public health interventions in Nicaragua are organized according to these official designations, we believe it is important to evaluate mosquito dynamics within that same classification system.

We agree that socio-economic conditions and infrastructure availability—particularly water supply reliability—are important drivers of *Aedes aegypti* productivity. However, our objective here was to provide a comparison between communities classified as rural and urban under the national surveillance framework while recognizing that these designations reflect a combination of factors, including infrastructure, housing density, and landscape structure. In this region of Managua, these elements interact to shape the ecological context in which *Ae. aegypti* populations develop.

To address the reviewer’s concern, we have revised the results to expand on more of the environmental and social aspects, as well as modified the discussion to clarify that the observed differences likely reflect a socio-ecological gradient rather than a purely land-cover distinction, and that infrastructure reliability and water storage practices are key mechanisms influencing mosquito production. At the same time, landscape configuration, housing density, and environmental structure remain important components of *Ae. aegypti* ecology in these settings. We have expanded the discussion to highlight these nuances and to acknowledge that the interpretation of “urban” and “rural” can vary globally depending on the operational definitions used by local public health authorities. In fact, this perspective aligns with an ongoing line of research within our group examining the integrated role of landscape, biological, and social determinants, we refer to this as the SCAPES framework, in shaping arbovirus vector ecology, for which additional analyses and manuscripts are currently in preparation.

**Conclusions**


-Are the conclusions supported by the data presented?

-Are the limitations of analysis clearly described?

-Do the authors discuss how these data can be helpful to advance our understanding of the topic under study?

-Is public health relevance addressed?

Reviewer #1: Again I am very satisfied with the authors efforts.

Reviewer #3: (No Response)

Reviewer #4: See methods/results comments

**Editorial and Data Presentation Modifications?**


Use this section for editorial suggestions as well as relatively minor modifications of existing data that would enhance clarity. If the only modifications needed are minor and/or editorial, you may wish to recommend “Minor Revision” or “Accept”.

Reviewer #1: Line 180. Prokopack aspirators instead of Prokopacks aspirators

**Response:** We have edited the text.

Reviewer #3: (No Response)

Reviewer #4: Standard errors of the mean should be reported with averages, not standard deviations

**Response:** We are now reporting SE with averages.

Table 2. sometimes a period is used for large numbers rather than a comma. Plus, this again is also a global comparison/description. The authors mention high heterogeneity of containers at a per house level. something to consider adding to this table is number of houses inspected, number of houses with positive containers. etc. - things like that

**Response:** We have changed all periods to commas and have added a column for Houses with containers. We did not add a column for number of inspected houses since it was always 250.

Figure 3 - I'm not sure how confidence bars nor significance is determined considering these would be values calculated across the entirety of the data. I'd also consider re-orienting this figure temporally rather than by dry/wet.

**Response:** Confidence intervals were removed from purely descriptive global indices to avoid inappropriate inferential interpretation.

Table 3: inside vs. outside? is this distinction made in any of the other analyses (for instance, is this considered in GLMMs/GAMMs). I have similar concerns for Figure 4 and 5.

**Response:** We clarify that indoor/outdoor aspiration was performed systematically; however, indoor/outdoor distinction was not included as a predictor in the primary models because total adult female counts per household were the response variable. We now clarify this methodological decision. Figure 4 and 5 have been collapsed to host all adult indices in a single figure.

Figure 6 is a plot of the smoothed function (not necessarily a prediction of the number of pupae compared to the number of adults). A prediction plot with observed data would be much more informative that this (which I would consider an internal diagnostic image of the smoothed function)

**Response:** We agree. The figure now includes:

- Panel A: Predicted adult female counts across pupal gradients with 95% CI and observed data overlay
- Panel B: Smoothed GAM function

**Summary and General Comments**


Use this section to provide overall comments, discuss strengths/weaknesses of the study, novelty, significance, general execution and scholarship. You may also include additional comments for the author, including concerns about dual publication, research ethics, or publication ethics. If requesting major revision, please articulate the new experiments that are needed.

Reviewer #1: I am very satisfied with the manuscript improvement and appreciate the authors efforts to address the reviewer concerns.

Minor issue which is completely optional. I'm opposed to the term "breeding sites" when describing larval habitats. Breeding is not happening in those sites, but larval development is. I recognize the term is common in the literature but consider making that change.

**Response:** We agree with the Reviewer and have changed the word breeding sites for larval development sites.

Reviewer #3: The authors have satisfactorily addressed the comments from the reviewers.

Reviewer #4: I think the original reviewers were correct to request more info on this being a rural vs. urban comparison. Global readers will think of this distinction as a function of economic drivers, when instead it appears more of a distinction of poverty. Much more context would be needed to explain this (pending the distinction even remaining if/when statistical modifications are made to further refine violations of independence at multiple levels within the data).

**Response:** We appreciate the Reviewer’s thoughtful observation regarding how the rural–urban distinction may be interpreted by a global audience. Our intention in using the rural and urban classification was to follow the operational categories defined by the Nicaraguan Ministry of Health (MOH), which guide vector surveillance and control activities in the country. Because public health interventions are organized according to these official designations, we considered it important to evaluate mosquito ecology within that same framework.

At the same time, we agree that differences between the study communities likely reflect a broader socio-ecological gradient that includes infrastructure reliability, water supply stability, housing characteristics, and container management practices. To address this concern, we have expanded the manuscript to provide additional context describing the structural and household characteristics of both communities (e.g., water supply interruptions, waste management, and housing conditions). These elements help clarify that the observed differences are not solely attributable to economic factors but rather reflect a combination of infrastructure access, water storage behavior, and environmental structure that together influence *Aedes aegypti* productivity.

Finally, we have revised the Discussion to acknowledge that the rural–urban classification in this study should be interpreted as part of a broader socio-ecological context, and we now explicitly discuss how infrastructure reliability and water storage practices contribute to the observed patterns of mosquito productivity. We believe these additions provide the necessary context for global readers while maintaining the relevance of the comparison within the local public health framework.
